# Immunohistochemical diagnosis of abdominal and lymph node tuberculosis by detecting *Mycobacterium tuberculosis *complex specific antigen MPT64

**DOI:** 10.1186/1746-1596-2-36

**Published:** 2007-09-25

**Authors:** Manju R Purohit, Tehmina Mustafa, Harald G Wiker, Odd Mørkve, Lisbet Sviland

**Affiliations:** 1 Centre for International Health, University of Bergen, Bergen, Norway; 2 Department of Pathology, Haukeland University Hospital, Bergen, Norway; 3 Department of Pathology, R.D. Gardi Medical College, Ujjain, India; 4 Section for Microbiology and Immunology, The Gade Institute, University of Bergen, Norway; 5 Department of Microbiology and Immunology, Haukeland University Hospital, Bergen, Norway; 6 Department of Thoracic Medicine, Haukeland University Hospital, Bergen, Norway

## Abstract

**Background:**

The aim of this study was to evaluate the diagnostic potential of immunohistochemistry using an antibody to the secreted mycobacterial antigen MPT64, in abdominal and lymph node tuberculosis.

**Methods:**

We used formalin-fixed histologically diagnosed abdominal tuberculosis (n = 33) and cervical tuberculous lymphadenitis (n = 120) biopsies. These were investigated using a combination of Ziehl-Neelsen method, culture, immunohistochemistry with an antibody to MPT64, a specific antigen for *Mycobacterium tuberculosis* complex organisms. Abdominal and cervical lymph node biopsies from non-mycobacterial diseases (n = 50) were similarly tested as negative controls. Immunohistochemistry with commercially available anti-BCG and nested PCR for IS6110 were done for comparison. Nested PCR was positive in 86.3% cases and the results of all the tests were compared using nested PCR as the gold standard.

**Results:**

In lymph node biopsies, immunohistochemistry with anti-MPT64 was positive in 96 (80%) cases and 4 (12.5%) controls and with anti-BCG 92 (76.6%), and 9 (28%) respectively. The results for cases and controls in abdominal biopsies were 25 (75.7%) and 2 (11.1%) for anti-MPT64 and 25 (75.7%) and 4 (22%) for anti-BCG. The overall sensitivity, specificity, positive and negative predictive values of immunohistochemistry with anti-MPT64 was 92%, 97%, 98%, and 85%, respectively while the corresponding values for anti-BCG were 88%, 85%, 92%, and 78%.

**Conclusion:**

Immunohistochemistry using anti-MPT64 is a simple and sensitive technique for establishing an early and specific diagnosis of *M. tuberculosis* infection and one that can easily be incorporated into routine histopathology laboratories.

## Background

Extra-pulmonary tuberculosis (EPTB) accounts for approximately 10–15% of all tuberculosis infections and occurs in up to 50% of patients with human immunodeficiency virus (HIV)-tuberculosis co-infection [1-3]. The annual incidence rates of EPTB have increased not only in developing countries but globally over the last few years [1,2,4].

The diagnosis of EPTB has always been problematical. Clinically, the disease presents in protean ways and histological examination is usually required for the diagnosis. Due to overlap of the histological features with other granulomatous conditions, the diagnosis of tuberculosis is dependent on the demonstration of acid fast bacilli (AFB) by Ziehl-Neelsen (ZN) staining. The yield of this method is limited however, in paucibacillary EPTB [5-7] and fresh unfixed tissue with live bacilli is usually not available for culture. Moreover, culture takes several weeks and is often negative in EPTB. There is therefore, a great need for a better diagnostic test to provide an alternative to AFB microscopy and culture.

While, *Mycobacterium tuberculosis *is the most common causative agent of EPTB, the prevalence of non-tuberculous mycobacteria is increasing with or without HIV infection and ranges from 3.8 to 50% in different parts of world [7-9]. As treatment is different for the two conditions, it is important to make a definitive diagnosis. However, in areas of the world where the disease is endemic, less than half receives an accurate diagnosis leading to inappropriate empirical treatment [10-12].

Detection of mycobacterial antigens by immunohistochemistry (IHC) using polyclonal and monoclonal antibodies is an alternative to conventional acid-fast staining. A large number of different mycobacterial antigens including BCG, lipoarabinomannan [5,13-17] have been detected with varying results in tissues. These are all common mycobacterial antigens and thus cannot discriminate *M. tuberculosis *from non-tuberculous mycobacteria. However, in a pilot study, we have recently described the high sensitivity and specificity of an in-house rabbit polyclonal antibody in biopsies from patients with tuberculous lymphadenitis to detect a secretory mycobacterial antigen, MPT64, which is present only in *M*. *tuberculosis *complex [14,18].

The present study was undertaken to further evaluate the diagnostic potential of immunohistochemial staining to detect MPT64 using a larger sample size from a different population and including other sites. Formalin fixed paraffin embedded biopsies from patients with abdominal tuberculosis and tuberculous lympadenitis were examined and the performance of anti-MPT64 was compared with the commercially available anti-BCG.

## Methods

Histologically diagnosed abdominal tuberculosis (n = 33) and cervical tuberculous lymphadenitis (n = 120) biopsies were obtained from the Department of Pathology, Ujjain Hospital, Ujjain, India between July 2004 to March 2006. The diagnostic categories included in the study for cases and controls are shown in table 1. Control biopsies were obtained from the Ujjain Hospital, and also the Department of Pathology, Haukeland University Hospital, Bergen, Norway. Two pulmonary tuberculosis biopsy specimens from the archive with numerous AFB on ZN staining were used as known positive control when required.

**Table 1:** Diagnostic categories of specimens tested.

Diagnosis	Cervical Lymph Nodes	Intestinal wall	Peritoneum	Mesentric Lymph Nodes*	Total number of specimens
**CASES**					
Tuberculosis	120	19	9	5	153

**NEGATIVE CONTROLS**					
Reactive/Non Specific inflammation	16	0	1	13	30
Foreign body granuloma#	10	0	0	0	10
Fungal granuloma	4	0	0	0	4
Parasitic granuloma	0	3	0	0	3
Malignancy	2	0	1	0	3

**TOTAL**	152	22	11	18	203

* no associated intestinal lesion seen.

# all foreign body granuloma were in skin biopsies.

Biopsies from patients with pulmonary tuberculosis, on corticosteroids, immunosuppressive therapy were excluded from the study. Detailed clinical history and examination results were obtained either from the clinical records or from the patients. The majority of lymphadenitis patients presented with a neck mass, while abdominal tuberculosis was mainly associated with abdominal pain. Informed written consent was obtained and the patients were ensured of confidentiality. Ethical approval was obtained from the Institutional ethical committee at Ujjain Hospital and the regional ethical committees in both Norway and India. All the patients were tested for HIV.

### Culture, ZN staining & Histopathology

One-half of the fresh biopsy specimens were submitted for mycobacterial culture on Lowenstein-Jensen egg media. The other half of the biopsy was fixed by 4% phosphate buffered formaldehyde for conventional paraffin embedding followed by routine haematoxylin and eosin and ZN stain to detect AFB. ZN staining was performed by heat carbol fuchsin method.

For histopathology, the sections were examined for the presence of granulomas and subdivided into two groups for analysis. Well-organized granulomas were characterized by a central group of epitheloid histiocytes, Langhan's giant cells, a mantle of lymphocytes and fibrous tissue. Poorly-organized granulomas showed a diffuse mixture of lymphocytes, histiocytes, and plasma cells with occasional giant cells. Each granuloma was also analyzed for the presence or absence of necrosis. The number of granulomas per sections, their type of organization and presence of necrosis was noted.

### Immunohistochemistry

IHC was performed using the DakoCytomation kit (EnVision + System-HRP; DakoCytomation Denmark A/S, Glostrup, Denmark). Tissue sections were deparaffinized, hydrated, and after microwave antigen retrieval, the endogenous peroxidase activity was inhibited by incubating the sections with hydrogen peroxide for 8 minutes. The slides were then treated with primary antibodies – (i) anti-BCG, (DAKO, Hamburg, Germany) at 1/5000 dilution for 1 hour after treating sections with 3% bovine serum albumin for 3 minutes, (ii) in-house absorbed polyclonal anti-MPT64 antibody at 1/250 dilution for 1 hour. Optimal dilutions were determined prior to these experiments. Sections were incubated with anti-rabbit dextran polymer conjugated to horseradish peroxidase for 45 minutes (30 minutes for anti-BCG). Antigen was visualized with 3-amino-9-ethylcarbazol- and hydrogen peroxide containing substrate and counter-stained with haematoxylin. All incubations were carried out at room temperature and the sections were thoroughly washed in-between incubations. In every experiment, one positive control and two negative controls were included. In one negative control primary antibody was substituted with antibody diluent and the other was with an irrelevant rabbit polyclonal antibody.

Mycobacterial antigen load was evaluated by counting the stained cells with light microscopy using a 40× ocular fitted with a 10 × 10 mm graticule and by evaluation of the staining intensity. For each section, three granulomas were selected for analysis. The number of stained epitheloid cells, stained giant cells and the total number of nucleated cells were counted for each granuloma and the results were presented as percentage of stained cells. The intensity of staining of section was evaluated separately and categorized as weak, moderate, and strong staining based on subjective assessment.

### Nested Polymerase Chain Reaction for IS6110

Five to six, 8 μm sections from each paraffin embedded tissue blocks were collected in sample preparation tubes for nested PCR. Carry-over tissue contamination was prevented by cleaning the blade with 96% ethanol after sectioning each sample; negative controls were sectioned first, followed by test blocks and positive control blocks.

DNA extraction and nested PCR on paraffin sections were performed as described previously [14]. Briefly, following proteinase K digestion, bacterial genomic DNA was eluted in water using a MagAttract DNA mini M48 Kit (Qiagen, West Sussex, UK) on Biorobot M48 (Qiagen). A 123-base pair fragment from IS6110 was amplified using the following primers 5' CCTGCGAGCGTAGGCGTCGG 3' and 5' CTCGTCCAGCGCCGCTTCGG 3'. The product was subjected to a second round of PCR amplification using the primers 5' TTCGGACCACCAGCACCTAA 3' and 5' TCGGTGACAAAGGCCACGTA 3' to amplify a 92-base pair fragment. The PCR reaction mixture consisted of 5 μl eluted DNA, 25 μl of HotStarTaq master mix (Qiagen), 0.25 μl of each 100 μM primer stock solution, distilled water to make a final volume of 50 μl. For nested PCR, 1 μl of the first PCR product was used as template. The reaction cycle for the first PCR was – 94°C for 1 minute, 68°C for 1 minute, 72°C for 20 seconds for 45 cycles and for the nested PCR – 94°C for 1 minute, 58°C for 1 minute, 72°C for 20 seconds for 35 cycles. Both PCR's had an initial heat activation step of 95°C for 15 minutes and a final extension of 72°C for 10 minutes. The amplified product was analyzed in a 3% agarose gel stained with ethidium bromide. Mycobacterial DNA, and positive PCR product were included as positive controls and an extraction control (with all the steps but without any tissue), a reaction tube with substitution of distilled water for the test template and a sample which previously yielded negative result on PCR were included as negative control in each PCR run.

### Data Analysis

Data entry and analysis was done using SPSS 12.0 for Windows. Pearsons chi-square test was used to determine the significance among categorical variables. Non-parametric tests were used for two-group comparisons. Differences were considered statistically significant if p ≤ 0.05. The diagnostic indices were calculated by decision matrix comparison.

## Results

A total of 203 biopsy specimens were studied. All the patients were negative for HIV.

### Acid-Fast Bacilli microscopy and Culture (table 2)

**Table 2:** Positive results of different diagnostic procedure on cervical lymph nodes and abdominal biopsies.

Diagnostic Procedure	Cervical Lymph Nodes Biopsy (n = 152)		Abdominal biopsy (n = 51)					
			
			Intestinal wall		Peritoneum		Mesenteric Lymph Nodes	
	
	Case (n = 120) n (%)	Control (n = 32) n(%)	Case (n = 19) n(%)	Control (n = 3) n(%)	Case (n = 9) n(%)	Control (n = 2) n(%)	Case (n = 5) n(%)	Control (n = 13) n(%)
ZN stain	14(11.6)	0	0	0	0	0	0	0
LJ Culture	27(22.5)	0	0	0	2(22)	0	2(40)	0
Anti-BCG	92(76.6)	9(28)	15(78.9)	1(33)	6(66)	1(50)	4(80)	2(15.4)
Anti-MPT64	96(80)	4(12.5)	14(73.7)	0	7(77.7)	1(50)	4(80)	1(7.7)
PCR	104(86.6)	3(9.4)	17(89)	0	7(77.7)	0	4(80)	1(7.7)

Acid fast bacilli were detected by ZN staining of abdominal and lymph node biopsies in 0/33 (0%) and 14/120 (11.7%) specimens respectively. *Mycobacterium *was isolated on culture from 4/33 (12.1%) and 27/117 (23%) specimens of abdominal and lymph node cases respectively. Culture results were not available from 3 cases and 24 controls. None of the control biopsies showed positive result for either of the tests.

### Histopathology

Both well-organized and poorly-organized granulomas were observed in biopsies from abdominal and lymph node cases. In abdominal tuberculosis, all the mesenteric lymph nodes showed well-organized necrotic granulomas and in intestinal wall, necrotic granulomas were seen in 58% of the cases while other cases had non-necrotic granulomas with or without fibrosis.

Lymph nodes showed typical well-organized granulomas in 70% of cases, mixed in 18% and poorly-organized in 12% of cases. Ninety percent of cases showed necrosis, however, both necrotic and non-necrotic granulomas were often seen in the same section.

### Immunohistochemistry

IHC with anti-MPT64 was positive in 14 (73.7%), 7 (77.7%) and 4 (80%) cases from intestinal wall, peritoneum, and mesenteric lymph node respectively and 2 (11%) controls. Similarly, the corresponding results for anti-BCG were 15 (78.9%), 6 (66%), 4 (80%) respectively for cases and 4 (22%) for controls (Table 2). In lymph node biopsies, IHC with anti-MPT64 was positive in 96 (80%) cases and 4 (12.5%) controls whereas anti-BCG was positive in 92 (76.6%) cases, and 9 (28.1%) controls.

**Figure 1:**
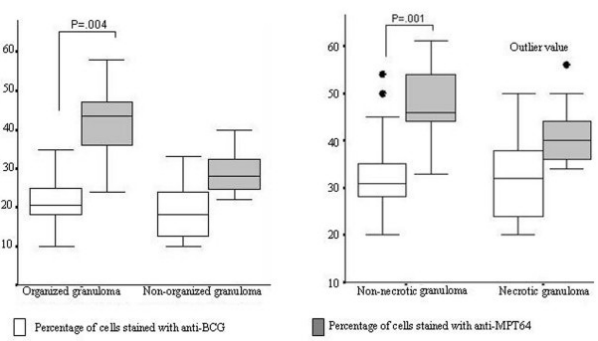
A: Percentage of stained cells by two antibodies in relation to organization of granuloma. B: Percentage of stained cells by two antibodies in relation to necrosis in granuloma.

**Figure 2:**
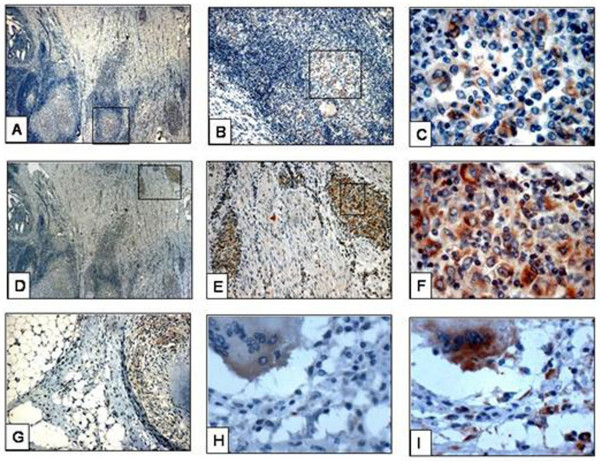
**Immunohistochemical staining in abdominal tuberculosis**. A-C : Staining by anti-BCG in granuloma in intestinal wall, D-F : staining by anti-MPT64 in granuloma in intestinal wall. The area in square is magnified in subsequent sections, G : staining by anti-BCG in granuloma in peritoneum, H : diffuse staining of giant cells in peritoneum by anti-BCG, I : same giant cell as shown in H showing strong, granular staining with anti-MPT64.

A comparison of the two antibodies looking at percentage of stained cells, and intensity of staining in relation to necrosis and organization of granulomas is shown in table 3 and figures 1 and 2. In the well-organized granulomas, the percentage of stained cells (p = 0·04) and the intensity of staining (+2 or +3; p = 0.001) was significantly higher for anti-MPT64 than anti-BCG. No significant difference of staining percentage or intensity was detected between the two antibodies in poorly-organized granulomas. In non-necrotic granuloma the percentage of positive cells (p = 0.02) and the intensity of staining (p = 0.03) was significantly higher with anti-MPT64 than with anti-BCG. In the necrotic granulomas, the intensity of staining with anti-MPT64 was higher but there was no significant difference between the percentages of stained cells between the two antibodies.

**Table 3:** Intensity of immunohistochemical staining by two antibodies in relation to granuloma features.

Granuloma characteristics	Intensity of anti-BCG staining			Intensity of anti-MPT64 staining		
	
	Mild	Moderate	Strong	Mild	Moderate	Strong
**Organization**						
Well Organised	46*	38	9	8	51**	34
Non Organised	28	8	1	13	14	10
**Necrosis**						
Non Necrotic	30	23	4	4	29	24
Necrotic	44	23	6	17	36***	20

*p = .02, **p = .001, ***p = .026

**Figure 3:**
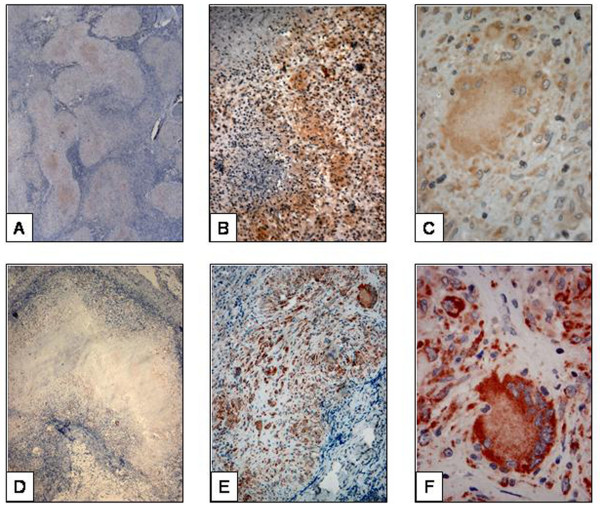
**Immunohistocheminal staining of lymph node tuberculosis**. A : Staining by anti-BCG in organized granuloma. The central necrotic area is also showing staining, B : staining by anti-BCG in poorly organized granuloma. There is diffuse staining with background staining, C : The diffuse and weak staining of giant cell by anti-BCG, D : staining by anti-MPT64 in organized granuloma. The central necrotic area is not showing staining, E : staining by anti-MPT64 in poorly organized granuloma. There is granular staining in clear background, F : The strong and granular staining of giant cell by anti-MPT64.

The location and pattern of expression of the two antibodies varied. Staining with anti-MPT64 was seen mainly in the inflammatory cells. The necrotic centres were generally negative, except in 8% of necrotic granulomas where occasional but strong signals were detected. Staining pattern was predominantly granular with anti-MPT64. With anti-BCG, positive signals were detected both in the necrotic centre and in inflammatory cells in all the necrotic granulomas. Unlike anti-MPT64, the staining pattern with anti-BCG was predominantly diffuse (fig. 2, 3).

The performance of the two antibodies in giant cells was assessed separately. Unlike the epithelioid cells, there was no significant difference in the percentage of giant cells stained with either of the antibodies. However, the intensity of staining for anti-BCG in the giant cells was weak compared to anti-MPT64 (fig. 2C, 2F).

### Polymerase Chain Reaction

The results of IS6110 PCR assay is shown in table 2. Overall PCR assay positivity was 132/153(86.6%) in histologcally diagnosed tuberculosis cases. Among these, eight cases were positive after the first PCR amplification while the majority (n = 124) were positive on nested PCR only. Cases which were positive with first PCR run had higher percentage of anti-MPT64 stained cells. All the positive controls were positive on both first PCR run and nested PCR.

### Comparison and validation of the result of various tests

The results of various tests were compared using nested PCR as the gold standard. All ZN and culture positive cases were positive for anti-MPT64, anti-BCG and PCR while ZN was positive in only 14 culture positive cases. With IHC using anti-MPT64 and anti-BCG positive results were found in125 and 120 specimens respectively with a sensitivity of more than 85% by both antibodies (table 4). Of 67 PCR negative cases, anti-MPT64 and anti-BCG were negative in 65 and 57 respectively, thus giving a specificity of 93% for anti-MPT64 and 88% for anti-BCG (table 4). Overall comparison showed significant differences in the sensitivity of IHC compared with sensitivity of ZN staining and culture (p =< 0.001). No significant difference was found in the specificity of IHC when compared with the other tests. The proportion agreement between PCR and anti-MPT64 was 93% (kappa-0.85).

**Table 4:** Diagnostic validation of different tests using nested PCR as gold standard (all values are in percentage).

Diagnostic method	Sensitivity	Specificity	PPV	NPV
**LYMPH GLAND TB**				
Anti-BCG	88	86	94	76
Anti-MPT64	93	98	99	85

**ABDOMINAL TB**				
Anti-BCG	86	81	86	81
Anti-MPT64	89	95	96	87

**TOTAL TB CASES**				
Anti-BCG	88	85	92	78
Anti-MPT64	92	97	98	85

PPV = positive predictive value, NPV = negative predictive value

### Comparison between Cervical Lymph Node and Abdominal Tissue Samples for various tests

In tuberculous lymphadenitis the sensitivity of ZN staining (13%) and culture (25%) was higher than in abdominal tuberculosis (0% and 12.5% respectively). The sensitivity of IHC with both the antibodies was high and there was no significant difference between cervical tuberculous lymph nodes and different sites of abdominal tuberculosis. The specificity of anti-BCG was, however, 60% in intestinal wall compared to 86% and 92.3% in cervical lymph nodes and mesenteric lymph nodes respectively. In contrast, specificity with MPT64 was very high and was found to be 100% in intestinal wall and mesenteric lymph nodes and 98% in cervical lymph nodes (table 4).

## Discussion

There have been several reports describing the use of IHC in the diagnosis of tuberculosis (14). However, in this study we show that using an antigen against the secretory mycobacterial antigen MPT64, it is possible to achieve consistently high sensitivity (89-93%) and specificity (95-98%) with IHC on different types of tissues. The strength of this technology is that it is robust, readily available in routine surgical pathology laboratories and can detect fragmented tubercle bacilli [19]. Compared with ZN staining that has a sensitivity of 10–45% [19] and requires an intact cell wall this technique offers a major improvement in diagnostic potential and should be suited for the diagnosis of pauci-bacillary EPTB. IHC for tuberculosis has, however, been slow to catch on as a routine diagnostic method in histopathology laboratories probably due to the lack of a specific anti-mycobacterial antibody suitable for all types of tissue [5,14,16] and hence the exact diagnostic role of IHC for *M. tuberculosis *has to be assessed in appropriate control groups and with appropriate antisera in endemic areas. Our large study is the first to show that IHC with an antibody to MPT64 is sufficiently robust to establish etiological diagnosis of *M*. *tuberculosis complex *infection in different types of tissues of EPTB.

Our results show that IHC with anti-MPT64 has better specificity, sensitivity, and predictive values than anti-BCG (table 4). This was particularly clear in intestinal wall tuberculosis, where anti-MPT64 showed a 100% specificity compared with 60% for anti-BCG (table 3). Anti-MPT64 antibodies also gave sharp and strong signals with clear background compared with anti-BCG antibodies making interpretation easier and permitting a more confident diagnosis of *M. tuberculosis *complex organism. Lower specificity with anti-BCG could be due to cross-reactivity with other infectious organisms as described earlier [20-22].

The sensitivity of anti-MPT64 is also very high but not very different from anti-BCG. The amplification method used in IHC improves the recognition of positive fragments. The few false negatives could be caused by the length of formalin fixation prior to processing which is known to reduce sensitivity[23]. Another explanation for the false negative results may be that the number of mycobacteria present is below the sensitivity level of IHC (about 5 × 10^5 ^to 1 × 10^6 ^organisms per gram tissue) and the lesions are exuberant inflammatory responses to a minimal number of organisms[24].

Four of the negative controls were positive with both PCR and IHC with both antibodies. Among these were two lymph node samples with histological changes of non-specific lymphadenitis that may represent early or latent tuberculosis infection. According to Goel *et al *[25], early tuberculous lesions may not be identified by histopathology because the formation of granulomas and emergence of the classical histopathological tuberculous picture may be a late phenomenon. They suggest that such cases might represent the transition between the incubation and development of disease[25]. Perhaps, in some cases, our IHC method can play a role in the early diagnosis of tuberculosis when histological examination fails to provide a diagnosis. The high prevalence of tuberculosis together with parasitic infection is well known in tuberculosis endemic countries. One of our controls with intestinal parasite turned out positive with IHC and PCR [26] and may well be a case of tuberculosis. It is difficult to explain the positive results of both tests on foreign body granulomas from Norway, however, as suggested by Mustafa *et al*, the possibility of latent infection cannot be ruled out [14].

In endemic countries, the majority of granulomatous lesions without necrosis are considered to be tuberculosis but this may not be the case in the developed world. Interestingly, when we looked specifically for the bacilli in the different zones of the granuloma they were more frequently detected by anti-MPT64 in the epitheloid cells than in the necrotic area. Using anti-BCG antibodies antigens were also detected in necrotic area. We also found that the percentage of stained cells was higher in non-necrotic granulomas than in necrotic granuloma with anti-MPT64 compared to anti-BCG with clearer and stronger signals. Hence, non-necrotic granulomatous lesion staining with MPT64 will support a diagnosis of tuberculosis.

Ideally, culture should be used as gold standard when comparing diagnostic test performance in tuberculosis. This investigation is, however, associated with low sensitivity especially in EPTB and as in our series, the corresponding results from controls are usually not available [27]. Histopathology remains one of the most important methods for diagnosing tuberculosis, however; it cannot differentiate changes caused by *M. tuberculosis*, non-tuberculous mycobacteria or other granulomatous diseases. We used nested PCR as the reference for comparison. In recent years, the sensitivity and specificity of PCR for diagnosis of tuberculosis has been well documented and is in the range of 60–98% in reported series where PCR was compared with culture as gold standard [28,29]. Our results also showed strong association between PCR and culture with all culture positive samples also being PCR positive. While PCR is increasingly used in the detection of mycobacteria from the tissue sample, the cost of the instruments and reagents, sensitivity to contamination and technical demand limits its use in developing countries [30].

## Conclusion

We have shown that IHC with an antibody to MPT64, a secreted antigen specific to the *M. tuberculosis *complex, is a specific and sensitive technique for diagnosis of EPTB. It is a cheap, robust and rapid method that can be used in a routine laboratory to provide a result in one working day and ensures the early institution of therapy. Being specific, anti-MPT64 would be of value in differentiating *M. tuberculosis *from other organisms, especially non-tuberculous mycobacteria, and other granulomatous inflammations.

## Competing interests

The author(s) declare that they have no competing interests.

## Authors' contributions

MRP, TM and LS designed the study, drafted the manuscript, and were also involved in the subject enrolment. MRP performed the experiments and data acquisition and analysis. All authors contributed, read and approved the final draft.
